# Patterns and Characteristics of Methamphetamine Use Among Adults — United States, 2015–2018

**DOI:** 10.15585/mmwr.mm6912a1

**Published:** 2020-03-27

**Authors:** Christopher M. Jones, Wilson M. Compton, Desiree Mustaquim

**Affiliations:** ^1^Office of Strategy and Innovation, National Center for Injury Prevention and Control, CDC; ^2^National Institute on Drug Abuse, National Institutes of Health, Bethesda, Maryland; ^3^Division of Overdose Prevention, National Center for Injury Prevention and Control, CDC.

Methamphetamine is a highly addictive central nervous system stimulant. Methamphetamine use is associated with a range of health harms, including psychosis and other mental disorders, cardiovascular and renal dysfunction, infectious disease transmission, and overdose (*1*,*2*). Although overall population rates of methamphetamine use have remained relatively stable in recent years (*3*), methamphetamine availability and methamphetamine-related harms (e.g., methamphetamine involvement in overdose deaths and number of treatment admissions) have increased in the United States* (*4*,*5*); however, analyses examining methamphetamine use patterns and characteristics associated with its use are limited. This report uses data from the 2015–2018 National Surveys on Drug Use and Health (NSDUHs) to estimate methamphetamine use rates in the United States and to identify characteristics associated with past-year methamphetamine use. Rates (per 1,000 adults aged ≥18 years) for past-year methamphetamine use were estimated overall, by demographic group, and by state. Frequency of past-year use and prevalence of other substance use and mental illness among adults reporting past-year use were assessed. Multivariable logistic regression examined characteristics associated with past-year use. During 2015–2018, the estimated rate of past-year methamphetamine use among adults was 6.6 per 1,000. Among adults reporting past-year methamphetamine use, an estimated 27.3% reported using on ≥200 days, 52.9% had a methamphetamine use disorder, and 22.3% injected methamphetamine. Controlling for other factors, higher adjusted odds ratios for past-year use were found among men; persons aged 26–34, 35–49, and ≥50 years; and those with lower educational attainment, annual household income <$50,000, Medicaid only or no insurance, those living in small metro and nonmetro counties,^†^ and those with co-occurring substance use and co-occurring mental illness. Additional efforts to build state and local prevention and response capacity, expand linkages to care, and enhance public health and public safety collaborations are needed to combat increasing methamphetamine harms.

Data are from 171,766 adults participating in the 2015–2018 NSDUHs, managed by the Substance Abuse and Mental Health Services Administration.^§^ NSDUHs collected information about the use of drugs, alcohol, and tobacco through in-person interviews with noninstitutionalized U.S. civilians aged ≥12 years. An independent, multistage area probability sample design for each state and the District of Columbia allows for production of national and state estimates. The average overall weighted response rate for the 2015–2018 NSDUHs was 51%. NSDUH variables included sex, age, race/ethnicity, urbanization status of county, education, annual household income, insurance status, and self-reported substance use, mental illness status, and receipt of substance use treatment. Self-reported substance use in NSDUHs included lifetime and past-year use of methamphetamine; past-year use of cocaine and heroin; past-year misuse of prescription opioids, sedatives, tranquilizers, and stimulants; past-month binge drinking (i.e., drinking five or more [men] or four or more [women] drinks on the same occasion on ≥1 day within the past month); and past-month nicotine dependence as determined using the Nicotine Dependence Syndrome Scale (*6*). NSDUHs assessed past-year substance use disorders for specific substances (e.g., methamphetamine) using self-reported responses to questions based on the individual diagnostic criteria from the *Diagnostic and Statistical Manual of Mental Disorders*, *Fourth Edition* (DSM-IV). Using a predictive model, past-year any mental illness and serious mental illness^¶^ were determined for each adult NSDUH respondent.

Using public-use-file data** from combined 2015–2018 NSDUHs, weighted counts, annual average rates per 1,000 adults, and corresponding 95% confidence intervals (CIs) were estimated for lifetime methamphetamine use and past-year methamphetamine use overall and by demographic, substance use, and mental illness variables. Estimates and 95% CIs for frequency of methamphetamine use and prevalence of past-year methamphetamine use disorder, methamphetamine injection, receipt of substance use treatment, other substance use, and mental illness among adults reporting past-year use were determined. Multivariable logistic regression examined characteristics associated with past-year methamphetamine use, controlling for demographic, substance use, and mental illness variables. Results are presented as adjusted odds ratios and 95% CIs. No multicollinearity or potential interaction effects between examined variables in the final model were observed. Restricted access 2017–2018 NSDUH data were used to estimate state rates of past-year methamphetamine use per 1,000 adults. NSDUHs use 2010 census-based population estimates (*3*). Stata (version 15.1; StataCorp) was used to account for the NSDUH complex survey design and sample weights.

During 2015–2018, the estimated annual average rate of lifetime methamphetamine use was 59.7 per 1,000 adults, or 14,686,900 adults on average each year. The estimated rate of past-year use was 6.6 per 1,000, or 1,626,200 adults on average each year (Table 1). Estimated rates of past-year use were 8.7 for men and 4.7 for women. The highest estimated rates were among adults aged 26–34 (11.0), 18–25 (9.3), and 35–49 (8.3) years and among non-Hispanic whites (7.5), Hispanics (6.7), and non-Hispanic other races (5.6). Estimated rates of past-year use also varied by the other demographic, substance use, and mental illness variables assessed. During 2017–2018 rates of past-year methamphetamine use ranged from 2.76 in New York to 13.98 in Nevada; generally, rates were higher in the western United States than in the East (Supplementary Figure, https://stacks.cdc.gov/view/cdc/85704).

**TABLE 1:** Methamphetamine use among adults aged ≥18 years by demographic, substance use, and mental health characteristics — United States, 2015–2018

Characteristic	Past-year methamphetamine use	
	Annual average no. of adults aged ≥18 years (weighted)	Annual average rate per 1,000 adults aged ≥18 years (95% CI)
**Overall lifetime use**	**14,686,900**	**59.7 (58.1–61.4)**
**Overall past-year use**	**1,626,200**	**6.6 (6.1–7.1)**
**Past-year use by demographic characteristic**		
**Sex**		
Women	598,300	4.7 (4.2–5.2)
Men	1,027,900	8.7 (7.9–9.5)
**Age group (yrs)**		
18–25	320,000	9.3 (8.3–10.4)
26–34	431,200	11.0 (9.7–12.5)
35–49	507,900	8.3 (7.3–9.5)
≥50	367,100	3.2 (2.8–3.9)
**Race/Ethnicity**		
White, non-Hispanic	1,180,200	7.5 (6.9–8.2)
Black, non-Hispanic	72,000	2.5 (1.8–3.4)
Other, non-Hispanic	113,000	5.6 (4.4–7.2)
Hispanic	260,900	6.7 (5.5–8.1)
**Education level**		
Less than high school diploma	394,600	12.4 (10.8–14.3)
High school graduate	563,300	9.2 (8.1–10.4)
Some college or associate’s degree	527,300	6.9 (6.1–7.9)
Bachelor’s degree or higher	141,000	1.8 (1.3–2.5)
**Annual household income**		
<$20,000	640,700	15.6 (13.8–17.7)
$20,000–49,999	552,000	7.6 (6.6–8.6)
$50,000–74,999	169,100	4.3 (3.4–5.5)
≥$75,000	264,300	2.9 (2.4–3.4)
**Insurance status**		
Private or other insurance (including Medicare)	704,900	3.6 (3.1–4.1)
Medicaid only	524,600	20.9 (18.5–23.5)
Uninsured	396,700	16.4 (13.9–19.2)
County type of residence*		
Large metro	711,200	5.2 (4.6–5.8)
Small metro	583,100	7.9 (6.6–9.5)
Nonmetro	331,900	9.5 (8.2–11.0)
**Substance use** ^†^		
Past-month binge drinking	753,900	11.6 (10.2–13.0)
Past-month nicotine dependence	719,900	39.0 (35.1–43.4)
Past-year marijuana use	1,118,000	30.6 (27.9–33.6)
Past-year cocaine use	493,500	94.7 (83.5–107.1)
Past-year heroin use	275,600	315.7 (267.8–367.8)
Past-year prescription opioid misuse	657,100	63.2 (56.4–70.9)
Past-year prescription sedative/tranquilizer misuse	473,400	74.0 (66.9–81.7)
Past-year prescription stimulant misuse	350,900	69.6 (61.2–79.0)
**Mental health**		
No past-year mental illness	688,300	3.4 (3.1–3.8)
Past-year mental illness but not serious mental illness^§^	531,900	15.3 (13.5–17.3)
Past-year serious mental illness^¶^	406,000	37.6 (32.1–43.9)

**Source:** National Surveys on Drug Use and Health, 2015–2018, using 2010 U.S. Censusꟷbased population estimates.

**Abbreviations:** CI = confidence interval; DSM-IV = *Diagnostic and Statistical Manual of Mental Disorders, Fourth Edition*.

* The Rural-Urban Continuum Codes are hierarchical, mutually exclusive classifications for all U.S. counties created by the U.S. Department of Agriculture. All population counts are from the 2010 Census representing the resident population. *Large metro* = counties in metro areas with a population ≥1 million persons. *Small metro* = counties in metros areas with populations between 250,000–1,000,000; counties in metro areas with populations <250,000. *Nonmetro* = counties with urban populations ≥20,000 adjacent to a metro area; urban populations ≥20,000 not adjacent to a metro area; urban populations 2,500–19,999 adjacent to a metro area; urban populations 2,500–19,999 not adjacent to a metro area; rural or <2,500 urban populations adjacent to a metro area; and rural or <2,500 urban population not adjacent to a metro area. https://seer.cancer.gov/seerstat/variables/countyattribs/ruralurban.html.

^†^ Among adults engaging in substance use behavior.

^§^ Any mental illness is defined as currently or at any time within the past year having had a diagnosable mental disorder (excluding developmental disorders and substance use disorders of sufficient duration to meet DSM-IV diagnostic criteria). https://www.samhsa.gov/data/report/2017-methodological-summary-and-definitions. For this analysis where the variable was defined as past-year mental illness, not serious mental illness, persons meeting criteria for serious mental illness were not included.

^¶^ Serious mental illness is defined as currently or at any time within the past year having had a mental disorder (excluding developmental disorders and substance use disorders of sufficient duration to meet DSM-IV diagnostic criteria), which resulted in serious functional impairment substantially interfering with or limiting one or more major life activities. https://www.samhsa.gov/data/report/2017-methodological-summary-and-definitions.

Among adults reporting past-year methamphetamine use, an estimated 36.2%, 19.2%, 17.2%, and 27.3% reported using methamphetamine 1–29 days, 30–99 days, 100–199 days, and ≥200 days, respectively; 22.3% reported injecting methamphetamine (Figure). Approximately one half (52.9%) of adults who reported past-year methamphetamine use met diagnostic criteria for past-year methamphetamine use disorder. Among those with past-year methamphetamine use disorder, an estimated 31.5% received any substance use treatment within the past year.

**FIGURE:**
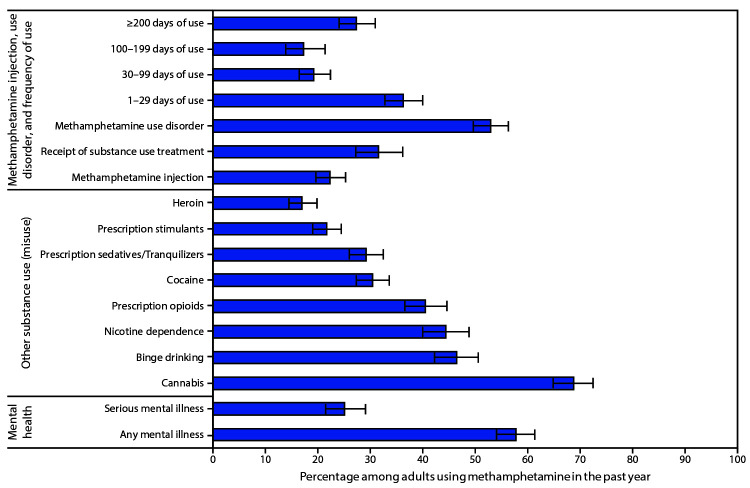
Methamphetamine injection, use disorder, frequency of use, receipt of substance use treatment,* other substance use,† and mental illness among adults aged ≥18 years reporting past-year methamphetamine use — United States, 2015–2018^§^ **Source:** National Surveys on Drug Use and Health, 2015-2018, using 2010 U.S. Censusꟷbased population estimates. * Receipt in past year among those with a methamphetamine use disorder; all other percentages are among adults reporting past-year methamphetamine use. ^†^ Binge drinking and nicotine dependence reported within the past month; all other substances are within the past year. ^§^ Weighted percentages; error bars represent 95% confidence intervals.

Among adults using methamphetamine within the past year, estimated prevalences of past-year use or misuse of other substances included cannabis use (68.7%), prescription opioid misuse (40.4%), cocaine use (30.4%), prescription sedative or tranquilizer misuse (29.1%), prescription stimulant misuse (21.6%), and heroin use (16.9%). Past-month binge drinking was reported by an estimated 46.4% and nicotine dependence by 44.3%. Mental illness was common also; of persons who used methamphetamine, an estimated 57.7% reported any mental illness, and 25.0% reported serious mental illness during the past year.

Multivariable logistic regression analysis found increased odds of past-year methamphetamine use among men; persons aged 26–34, 35–49, and ≥50 years (versus persons aged 18–25 years); persons with less than a high school diploma, a high school diploma, and some college or associate’s degree (versus college graduates); those with annual household income <$20,000 or $20,000–$49,999 (versus ≥$75,000); persons having Medicaid only or being uninsured (versus private or other insurance); persons living in small metro and nonmetro counties (versus large metro counties); persons reporting past-month nicotine dependence; those reporting past-year use of cannabis, cocaine, and heroin; persons reporting misuse of prescription opioids, sedatives, tranquilizers, or stimulants; and persons reporting past-year mental illness but not serious mental illness or past-year serious mental illness (versus no past-year mental illness). Non-Hispanic black race/ethnicity was associated with lower odds of past-year methamphetamine use compared with non-Hispanic white race/ethnicity (Table 2).

**TABLE 2:** Characteristics associated with past-year methamphetamine use among adults aged ≥18 years — United States, 2015–2018

Characteristic	Adjusted odds ratios* (95% CI)
**Sex**	
Women	Reference
Men	1.68 (1.43–1.96)
**Age group (yrs)**	
18–25	Reference
26–34	1.67 (1.36–2.05)
35–49	2.49 (2.01–3.07)
≥50	1.72 (1.31–2.25)
**Race/Ethnicity**	
White, non-Hispanic	Reference
Black, non-Hispanic	0.29 (0.20–0.42)
Other, non-Hispanic	1.07 (0.78–1.47)
Hispanic	1.08 (0.85–1.37)
**Education level**	
Less than high school	3.28 (2.13–5.06)
High school graduate	2.65 (1.78–3.93)
Some college or associate’s degree	2.04 (1.38–3.02)
Bachelor’s degree or higher	Reference
**Annual household income**	
<$20,000	2.09 (1.59–2.74)
$20,000–49,999	1.42 (1.11–1.82)
$50,000–74,999	1.06 (0.77–1.46)
≥$75,000	Reference
**Insurance status**	
Private or other insurance (including Medicare)	Reference
Medicaid only	2.01 (1.55–2.61)
Uninsured	1.70 (1.31–2.22)
**County type of residence^†^**	
Large metro	Reference
Small metro	1.32 (1.01–1.72)
Nonmetro	1.54 (1.25–1.90)
**Substance use^§^**	
Past-month binge drinking	1.06 (0.86–1.30)
Past-month nicotine dependence	2.14 (1.75–2.62)
Past-year cannabis use	4.61 (3.67–5.80)
Past-year cocaine use	2.72 (2.12–3.50)
Past-year heroin use	5.10 (3.63–7.17)
Past-year prescription opioid misuse	2.17 (1.66–2.84)
Past-year prescription sedative/tranquilizer misuse	1.85 (1.45–2.35)
Past-year prescription stimulant misuse	1.91 (1.43–2.55)
**Mental health**	
No past-year mental illness	Reference
Past-year mental illness but not serious mental illness^¶^	2.18 (1.82–2.60)
Past-year serious mental illness**	3.34 (2.53–4.40)

**Source:** National Surveys on Drug Use and Health, 2015–2018, using 2010 U.S. Censusꟷbased population estimates.

**Abbreviations:** CI = confidence interval; DSM-IV = *Diagnostic and Statistical Manual of Mental Disorders, Fourth Edition*.

* Odds ratios are adjusted for all other variables in the model.

^†^ The Rural-Urban Continuum Codes are hierarchical, mutually exclusive classifications for all U.S. counties created by the U.S. Department of Agriculture. All population counts are from the 2010 Census representing the resident population. *Large metro* = counties in metro areas with a population ≥1 million persons. *Small metro* = counties in metros areas with populations between 250,000–1,000,000; counties in metro areas with populations <250,000. *Nonmetro* = counties with urban populations ≥20,000 adjacent to a metro area; urban populations ≥20,000 not adjacent to a metro area; urban populations 2,500–19,999 adjacent to a metro area; urban populations 2,500–19,999 not adjacent to a metro area; rural or <2,500 urban populations adjacent to a metro area; and rural or <2,500 urban population not adjacent to a metro area. https://seer.cancer.gov/seerstat/variables/countyattribs/ruralurban.html.

**^§^** Reference group is no use (misuse) within the past month (past year).

^¶^ Any mental illness is defined as currently or at any time within the past year having had a diagnosable mental disorder (excluding developmental disorders and substance use disorder of sufficient duration to meet DSM-IV diagnostic criteria). https://www.samhsa.gov/data/report/2017-methodological-summary-and-definitions. For this analysis where the variable was defined as past-year mental illness, not serious mental illness, persons meeting criteria for serious mental illness were not included.

** Serious mental illness is defined as currently or at any time within the past year having had a mental disorder (excluding developmental disorders and substance use disorder of sufficient duration to meet DSM-IV diagnostic criteria), which resulted in serious functional impairment substantially interfering with or limiting one or more major life activities. https://www.samhsa.gov/data/report/2017-methodological-summary-and-definitions.

## Discussion

In the United States during 2015–2018, approximately 1.6 million adults, on average, used methamphetamine each year, and nearly 25% of those reported injecting methamphetamine. In addition, approximately 50% of persons using methamphetamine in the past year met diagnostic criteria for past-year methamphetamine use disorder, yet fewer than one third of adults with past-year methamphetamine use disorder received substance use treatment in the past year. Particularly concerning were high rates of co-occurring substance use or mental illness among adults using methamphetamine.

These findings provide new insights into populations to prioritize for prevention and response efforts, such as men, middle aged adults, and rural residents. Identification of higher rates of methamphetamine use in small metro and nonmetro areas are important given difficulties in delivering services to rural populations who might be disproportionately affected by methamphetamine use. Attention has been drawn to infectious disease transmission associated with opioid injection in these areas (*7*); the long-standing challenges with lower economic resources, prevalent substance use, and limited treatment availability also place these areas at risk for infectious disease outbreaks associated with methamphetamine injection. Expansion of evidence-based substance use treatment, syringe services programs, and other community-based interventions aimed at reducing use, including injection, are needed.

Given the high rates of co-occurring substance use identified, along with trends of increasing opioid-related overdose deaths and treatment admissions that involve methamphetamine (*4*,*5*), prevention and treatment efforts will need to be comprehensive and broad-based. Universal preventive interventions such as Promoting School-Community-University Partnerships to Enhance Resilience (PROSPER) have resulted in lasting protective effects on youth substance use generally, and for methamphetamine use and opioid misuse specifically (*8*). Promising treatment strategies for methamphetamine use disorder are those that use evidence-based psychosocial approaches (e.g., community reinforcement or cognitive-behavioral therapy) combined with contingency management, where rewards are provided to reinforce positive behavior (*9*). The finding of increased odds of methamphetamine use among adults with lower socioeconomic indicators underscores the importance of recovery support services and linkage to social service providers.

The overlap of methamphetamine use with mental illness, especially serious mental illness, suggests an important role for mental health providers to engage in care with this population, in coordination with addiction and other health care providers. Treatment of co-occurring mental and substance use disorders has been a recognized gap in the system of care (*10*) and persons who use methamphetamine might be particularly affected.

The findings in this report are subject to at least four limitations. First, NSDUH data are self-reported and subject to recall and social desirability biases. Second, because the survey is cross-sectional and different persons were sampled each year, inferring causality from the observed associations between the predictors examined and self-reported past-year methamphetamine use is not possible. Third, NSDUHs do not include homeless persons not living in shelters, active duty military, or persons residing in institutions such as those who are incarcerated; thus, substance use estimates in this study might not be generalizable to the total U.S. population. Finally, NSDUHs provide estimates of persons meeting diagnostic criteria for methamphetamine use disorder based on self-reported responses to the individual questions that make up the DSM-IV diagnostic criteria for methamphetamine use disorder, not estimates of the number of persons receiving a diagnosis from a health care provider; thus, gaps between meeting diagnostic criteria and receiving treatment might be incorrectly estimated.

Methamphetamine use and related harms represent a substantial U.S. public health concern. Additional efforts to support prevention and response capacity in communities, expand linkages to care for substance use and mental health, and enhance collaborations between public health and public safety are needed.

**Summary**

**What is already known about this topic?**

Methamphetamine is a highly addictive central nervous system stimulant. In recent years, methamphetamine availability and methamphetamine-related harms have been increasing in the United States.

**What is added by this report?**

During 2015–2018, an estimated 1.6 million U.S. adults aged ≥18 years, on average, reported past-year methamphetamine use; 52.9% had a methamphetamine use disorder, and 22.3% reported injecting methamphetamine within the past year. Co-occurring substance use and mental illness were common among those who used methamphetamine within the past year.

**What are the implications for public health practice?**

Efforts to build state and local prevention and response capacity, expand linkages to care, and enhance public health and public safety collaborations are needed to combat rising methamphetamine availability and related harms.

## Footnotes

* https://www.nflis.deadiversion.usdoj.gov/DesktopModules/ReportDownloads/Reports/12568NFLISdrugMethamphetamine.pdf.

† The Rural-Urban Continuum Codes are hierarchical, mutually exclusive classifications for all U.S. counties created by the U.S. Department of Agriculture. All population counts are from the 2010 Census representing the resident population. *Large metro* = counties in metro areas with a population ≥1 million persons. *Small metro* = counties in metros areas with populations between 250,000–1,000,000; counties in metro areas with populations <250,000. *Nonmetro* = counties with urban populations ≥20,000 adjacent to a metro area; urban populations ≥20,000 not adjacent to a metro area; urban populations 2,500–19,999 adjacent to a metro area; urban populations 2,500–19,999 not adjacent to a metro area; rural or <2,500 urban populations adjacent to a metro area; and rural or <2,500 urban population not adjacent to a metro area. https://seer.cancer.gov/seerstat/variables/countyattribs/ruralurban.html.

§ https://www.samhsa.gov/data/report/2017-methodological-summary-and-definitions.

¶ Any mental illness is defined as currently or at any time within the past year having had a diagnosable mental disorder (excluding developmental disorders and substance use disorder) of sufficient duration to meet DSM-IV diagnostic criteria. Serious mental illness is defined as currently or at any time within the past year having had a mental disorder (excluding developmental disorders and substance use disorder) of sufficient duration to meet DSM-IV diagnostic criteria, which resulted in serious functional impairment substantially interfering with or limiting one or more major life activities. https://www.samhsa.gov/data/report/2017-methodological-summary-and-definitions.

** https://datafiles.samhsa.gov/info/browse-studies-nid3454.
